# A Low-Cost Modular Multi-Region Electrode for Distributed Network Recording and Brain State Decoding

**DOI:** 10.3390/brainsci16060606

**Published:** 2026-06-01

**Authors:** Bo-Yu Wang, Yu Chen, Bin Wang, Wen Xie, Jia-Yi Zeng, Yi-Zheng Wang, Chun-Kui Zhang

**Affiliations:** 1 Center of Cognition and Brain Science, Beijing Institute of Basic Medical Sciences, Beijing 100850, China; wangby0521@163.com (B.-Y.W.); chenyu0450@foxmail.com (Y.C.); xiechuanbin2003@163.com (B.W.); xiew021@163.com (W.X.); 18673858598@163.com (J.-Y.Z.); 2 Hengyang Medical School, University of South China, Hengyang 421001, China; 3 State Key Laboratory of Medical Neurobiology, National Clinical Research Center for Aging and Medicine, Huashan Hospital, Fudan University, Shanghai 200040, China

**Keywords:** modular electrode, multi-region, local field potential

## Abstract

Background: Precise decoding of brain states is essential for closed-loop neuromodulation, but current electrodes and recording strategies are inadequate. Multi-region recording offers network-level advantages over single-region approaches, yet remains underdeveloped due to the lack of low-cost, flexible multi-region electrodes and standardized workflows. Methods: We developed a low-cost, modular, silica capillary tube-based 16-channel electrode for multi-region local field potential (LFP) recording in small animals, along with an integrated workflow for spectral analysis, functional connectivity assessment, and machine learning-based brain state decoding. Results: Our electrode design enables flexible customization of target regions and low-cost (~19.43 USD/unit) assembly without specialized equipment. In vivo validation in rats targeting eight emotional network nuclei achieved 79.2% implantation accuracy, with stable LFP recordings maintained for over 3 months. In a reserpine-induced depression model, spectral analysis revealed state-specific oscillatory changes including reduced alpha power in the infralimbic cortex. Inter-regional functional connectivity analysis further captured drug-induced network-level synchronization changes. We also developed a machine learning pipeline with random forest classifier that achieved ~96.4% accuracy in decoding brain states from the multi-region signals. Conclusions: This modular multi-region electrode provides a practical, adaptable, and low-cost platform for long-term distributed network recording, supporting quantitative LFP analysis, functional connectivity assessment, and high-accuracy brain state decoding, laying a technical foundation for preclinical closed-loop neuromodulation research.

## 1. Introduction

Deep brain stimulation (DBS) has emerged as a core therapeutic strategy for neurological disorders such as Parkinson’s disease (PD) and epilepsy, and a promising investigational approach for psychiatric disorders including depression [1,2,3,4]. With advances in brain science and engineering, the field is increasingly moving toward closed-loop neuromodulation, a paradigm that integrates real-time neural signal feedback to dynamically adjust stimulation parameters, markedly improving therapeutic specificity and reducing side effects [5,6,7]. However, the successful implementation of this paradigm hinges on robust, stable disease-related biomarkers, and the identification and validation of such biomarkers remain major translational challenges [5,8].

Growing evidence confirms that abnormal inter-regional brain interactions are not only core manifestations of many neurological and psychiatric disorders but also critical potential biomarkers for closed-loop modulation [8]. Compared with single-region recording, multi-region neural signal analysis offers distinct advantages for predicting symptoms and elucidating disease mechanisms [9]. For PD, excessive β-band oscillations (13–30 Hz), are consistently observed across the basal ganglia, primary motor cortex and thalamus, which are closely linked to cardinal motor symptoms [10,11,12]. In depression, dysregulated connectivity between the prefrontal cortex and amygdala, along with signal alterations linked to hippocampal atrophy, are widely recognized as core pathological features [13,14,15]. These network-level abnormalities underscore the limitations of single-region recordings, making multi-region simultaneous recording essential for decoding brain network connectivity, investigating disease pathogenesis, and advancing closed-loop neuromodulation.

However, current electrodes used in preclinical research are insufficient to meet these demands, as they suffer from inherent limitations that hinder multi-region network recording. Clinically, electroencephalography (EEG) suffers from low spatial resolution [16], while electrocorticography (ECoG) and stereoelectroencephalography (sEEG) offer improved spatial precision but are constrained by fixed electrode layouts, which cannot flexibly adapt to individualized multi-region targeting [17,18]. In preclinical studies, traditional microwire bundles lack scalability for distributed networks [19], whereas emerging silicon-based microelectrodes and flexible electrodes prioritize spike recording, and require complex, costly microfabrication [20]. Additionally, most such electrodes adopt fixed structural designs, making them difficult to adjust implantation strategies flexibly to meet specific experimental requirements [21].

To address these limitations, building on and optimizing existing electrodes used in preclinical research, this study developed a modular, silica capillary tube-based 16-channel recording system for distributed brain network recording in small animals. Unlike existing devices that prioritize spike activity, adopt fixed layouts, or require costly fabrication, our design focuses on stable, long-term LFP acquisition via a simplified manufacturing process that does not require specialized equipment. It enables flexible customization of brain region combinations and implantation coordinates to meet diverse experimental goals, overcoming key constraints of conventional commercial arrays. To analyze multi-region LFP signals, we adopted an analytical workflow adapted from neuroimaging fields, including spectral feature extraction, feature selection, machine learning decoding, to process multi-region LFP signals. This integrated toolset supports reliable, long-term synchronous multi-region recording and quantitative network analysis, providing a technical foundation for preclinical mechanistic research and accelerating the translation of closed-loop neuromodulation.

## 2. Materials and Methods

### 2.1. Fabrication of Multi-Region Recording Electrodes

A custom-printed circuit board (PCB) with a double-sided green substrate was designed with 20 through-hole perforations (finished hole diameter: 0.3 mm, pitch: 1.27 mm). The board dimensions are 13.5 mm × 6 mm × 1 mm, and each pad measures 0.6 mm × 2.5 mm. Among the 20 holes, 16 were allocated for recording channels and 4 for ground and reference connections. A 2 × 10-pin double-row female header connector (1.27 mm pitch, Shenzhen Nanmenzi Technology Co., Shenzhen, China) was soldered to the PCB according to the predefined circuit layout.

Tungsten wires (California Fine Wire Company, Grover Beach, CA, USA; bare conductor diameter: 0.0013 inches (~33 μm), coated with a 5 μm-thick polyimide insulating layer, total outer diameter: ~43 μm) were cut into equal-length segments (60 mm). Each tungsten wire segment was inserted into a perforation on the PCB, followed by insertion of a custom-fabricated gold pin (Creation Tech, Beijing, China; GP-100) into the same hole. The pins were designed with the following dimensions: an overall length of 2.25 mm, with a 0.50 mm-diameter head; the conical shank had a base diameter of 0.40 mm, a tip diameter of 0.20 mm. The gold pin mechanically secured the tungsten wire and penetrated its insulating layer, establishing stable electrical contact between the wire and the PCB. This process was repeated for all 16 recording channels. For the four ground and reference perforations, 0.1 mm silver wires (Kunshan High-Purity Electronic Materials Business Department, Kunshan, China) were soldered directly into the holes.

Every two tungsten wires were grouped into one bundle, and each bundle was inserted into a 30 mm silica capillary tube (Polymicro Technologies, Phoenix, AZ, USA; TSP100170; inner diameter: 100 μm, outer diameter: 164 μm). To ensure consistent recording quality, the distal tip of each wire bundle was trimmed under a stereomicroscope using fine surgical scissors to a fixed 0.5 mm exposure length beyond the silica capillary tube, producing clean, blunt-end terminations. During this trimming process, the polyimide insulation at the distal tip was mechanically removed by the cutting action, leaving only the bare tungsten conductor exposed. No additional electrochemical treatment, polishing, or manual stripping of the insulation was performed. This standardized tip preparation enabled effective neural signal detection while minimizing tissue damage. After assembly of all wire bundles, all solder joints and interconnection interfaces on the PCB were fully encapsulated with insulating adhesive to prevent cross-channel interference and short circuits.

Electrical impedance of each channel was measured individually using the CerePlex Direct system (Blackrock Neurotech, Salt Lake City, UT, USA). Electrodes with impedance values outside the predefined acceptable range (100–1000 kOhm) were discarded. Qualified electrodes were labeled to establish a clear mapping between each channel, the acquisition system, and the target brain regions for subsequent implantation.

### 2.2. Experimental Animals

A total of 6 male Sprague-Dawley (SD) rats, weighing 280–320 g, were used in this study. The rats were housed in a controlled environment with a temperature ranging between 20 °C and 22 °C and a 12 h light/dark cycle. They were housed at a density of 2 rats per cage, with free access to standard chow and drinking water. Penicillin (80,000 IU/day, i.p.) was administered for the first 3 postoperative days to prevent infection. The experiment was approved by the Institutional Animal Care and Use Committee of the Beijing Institute of Basic Medical Sciences and strictly followed the 3R principles of animal welfare.

### 2.3. Electrode Implantation Surgery

Rats were anesthetized with sodium pentobarbital (30 mg/kg, intraperitoneal injection) and fixed in a stereotaxic instrument (RWD Life Science Co., Ltd., Shenzhen, China). The scalp was incised and retracted to expose the skull, and the skull surface was cleaned to remove the periosteum. A small hole was drilled at the preset position according to the stereotaxic coordinates. The silica capillary tube of the self-made 16-channel electrode (composed of 8 bundles) was clamped with a specialized electrode holder to facilitate stereotaxic manipulation.

Stereotaxic coordinates of each brain region were determined with reference to the Paxinos and Watson Rat Brain Atlas, with the bregma as the origin [22]. The specific coordinates (AP: anterior–posterior, ML: mediolateral, DV: dorsoventral) were as follows: Prelimbic cortex (PrL): AP: +3.24 mm, ML: +0.6 mm, DV: −3.6 mm; In-fralimbic cortex (IL): AP: +3.24 mm, ML: +0.6 mm, DV: −5.0 mm; Nucleus Accumbens shell (Nac): AP: +1.0 mm, ML: +2.9 mm, DV: −8.0 mm; Basolateral Amygdala (BLA): AP: −2.28 mm, ML: +5.0 mm, DV: −8.6 mm; dorsal Hippocampus (dHPC): AP: −3.24 mm, ML: +2.3 mm, DV: −3.2 mm; Ventral Tegmental Area (VTA): AP: −5.6 mm, ML: +0.4 mm, DV: −8.3 mm; Periaqueductal Gray (PAG): AP: −7.56 mm, ML: +0.8 mm, DV: −5.7 mm; and ventral Hippocampus (vHPC): AP: −5.6 mm, ML: +4.8 mm, DV: −7.4 mm. The spatial layout of these brain regions was distributed in the same hemisphere, and each electrode bundle (two channels per bundle) was assigned to one brain region, forming a clear 8-bundle (16-channel) mapping relationship for subsequent implantation.

Each electrode bundle was slowly inserted into the corresponding target brain region along the drill hole and the silica capillary tube was fixed to the skull with tissue adhesive, followed by the sequential implantation of the remaining bundles. After confirming the insertion position of all bundles, the electrode base was fixed to the skull with dental cement. Rats were placed in a warm environment to recover from anesthesia, and were monitored daily during the 7-day postoperative recovery period. Wound healing status and activity behavior were observed to exclude abnormal responses caused by surgery or electrode implantation.

### 2.4. Neural Signal Acquisition Under Free Movement

Seven days after surgery, the CerePlex Direct system was connected to the implanted electrode to establish a stable signal link. The system parameters were set as follows: sampling rate: 1 kHz, filtering range for local field potential (LFP): 0.1–300 Hz. Rats were placed in a free movement recording box (50 × 50 × 40 cm), and synchronous signal acquisition of 8 brain regions (16 channels) was performed. During recording, the behavior of rats was observed in real time to ensure no abnormal activities (such as restlessness, scratching the electrode, or movement limitation) caused by the electrode or the system connection line. The acquisition process was monitored continuously to avoid signal interruption. After acquisition, the signal data were exported and preprocessed to remove artifacts caused by external electrical interference. To record neural signals under post-reserpine conditions, rats were administered a single intraperitoneal (i.p.) injection of reserpine at a dose of 4 mg/kg. Reserpine was dissolved in a mixed solvent of DMSO, polyethylene glycol 300 (PEG300), Tween 80 and normal saline at a volume ratio of 1:4:0.5:4.5. Baseline neural signals were recorded before drug administration. Post-treatment recordings were performed 2 h after injection.

### 2.5. Verification of Implantation Position Accuracy

After the completion of signal recording, rats were euthanized by excessive anesthesia and perfused transcardially with normal saline followed by 4% paraformaldehyde. Brains were removed and fixed in 4% paraformaldehyde for 24 h, then dehydrated in a graded sucrose series (10%, 20%, 30%). Coronal frozen sections were cut on a freezing microtome (Leica Microsystems, Wetzlar, Germany) at a thickness of 30 μm, and sections were mounted on glass slides and subsequently covered with DAPI-containing fluorescent mounting medium (1:1000 dilution). The implantation trajectory of each electrode bundle was then observed using the Fully Automatic Virtual Digital Slide Scanning System (Olympus, Hachioji, Japan) to verify the accuracy of the implantation position relative to the target brain regions.

### 2.6. LFP Signal Preprocessing

Preprocessing of the acquired LFP signals involved multiple stages: removal of powerline interference using 50 Hz and 100 Hz notch filters, application of a 0–130 Hz band-pass filter, and segmentation into consecutive 2 s epochs. Artifact-contaminated epochs were omitted. Specifically, epochs were rejected if the peak-to-peak amplitude exceeded 1000 μV in any channel, or if visual inspection identified prominent movement artifacts or cable-induced noise transients. A region-wise average of the two corresponding channels was computed to produce a unified signal per target area. For each epoch, spectral analysis was conducted using Welch’s method to obtain PSD estimates [23]. These estimates were averaged within five canonical frequency bands—delta (0–4 Hz), theta (4–8 Hz), alpha (8–13 Hz), beta (13–30 Hz), and gamma (30–80 Hz). The resultant band-specific total power values were then employed as key features in all subsequent analyses.

### 2.7. Statistical Analyses and Machine Learning

Statistical analysis was performed using the Wilcoxon signed-rank test [24] to compare power spectral density (PSD) values across each frequency band and recording channel. This test provides a nonparametric alternative to the paired *t*-test and is appropriate for assessing differences between two related samples without assuming normality. A total of six rats were included in the analysis.

To evaluate the statistical significance of feature distribution differences between the baseline and reserpine-treated conditions, a two-tailed Mann–Whitney U test [25] was performed on each feature. Missing values were excluded prior to analysis to preserve data integrity and ensure valid statistical inference.

For brain state classification, all 40 features (8 channels × 5 frequency bands) were used directly without prior feature selection to avoid data leakage. A binary random forest classifier was constructed using the full 40-dimensional feature vectors for each animal independently, and model performance was evaluated via five-fold cross-validation, with Z-score normalization parameters computed exclusively on the training set of each fold and then applied to the corresponding test set. Accuracy, precision, recall, F1-score, and ROC-AUC were adopted as core evaluation metrics.

To investigate inter-regional functional connectivity, LFP signals from each epoch were first bandpass-filtered into the five canonical frequency bands (delta, theta, alpha, beta, gamma) using a fourth-order zero-phase Butterworth filter. For each frequency band, Pearson correlation coefficients were computed between all 28 pairs of the eight target brain regions, yielding an 8 × 8 functional connectivity matrix per epoch. These matrices were averaged across epochs within each condition (baseline and post-reserpine) to obtain condition-specific mean connectivity profiles. Difference matrices were then calculated by subtracting the baseline connectivity matrix from the post-reserpine matrix (Δ = reserpine − baseline), thereby quantifying drug-induced changes in inter-regional synchronization. Due to the limited sample size, the connectivity analysis was performed on a representative individual animal as an illustrative demonstration of the electrode system’s capability for network-level analysis.

### 2.8. Analysis Environment

All signal processing and statistical analyses were performed in the Python 3.10 computing environment. Core open-source libraries utilized included MNE-Python for the import, preprocessing, and spectral decomposition of local field potential (LFP) signals; SciPy for digital filtering, Welch’s power spectral density estimation, and nonparametric statistical tests; and scikit-learn for feature normalization, ANOVA-based supervised feature selection, binary classification, five-fold cross-validation, and the computation of key performance metrics such as accuracy, precision, recall, F1-score, and ROC-AUC.

## 3. Results

### 3.1. Design and Configuration of the Modular Silica Capillary Tube-Based Electrode

**Figure 1:**
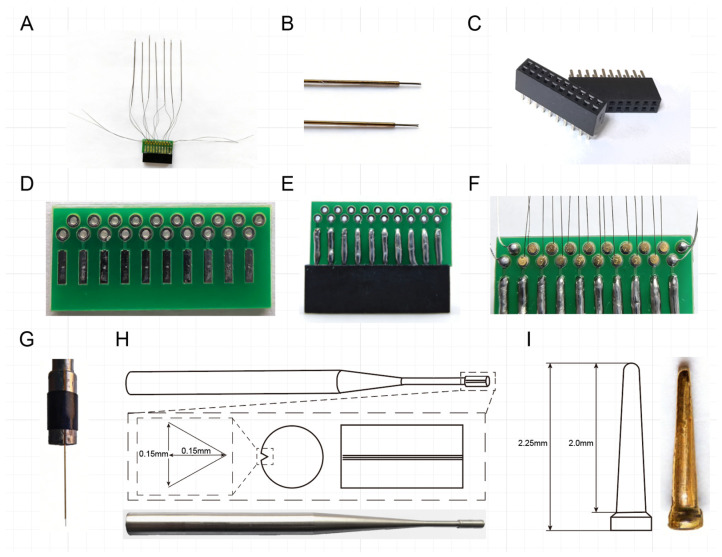
Fabrication components, assembly structure, and supporting implantation tools of the 16-channel modular electrode. (**A**) Photograph of fully assembled 16-channel modular electrodes, consisting of 8 independent electrode bundles with two tungsten wires in each bundle and 4 silver wires used as ground and reference electrodes, which are integrated on the printed circuit board (PCB) base. Among them, the two electrode bundles targeting the prelimbic cortex (PrL) and infralimbic cortex (IL) have identical anteroposterior (AP) and mediolateral (ML) stereotaxic coordinates, and only differ in dorsoventral (DV) implantation depth. They are bonded side by side into one bundle for easier implantation. (**B**) Electrode bundle encapsulated in a silica capillary tube, with the tungsten wire tips uniformly exposed by 0.5 mm. (**C**) Commercial 2 × 10 pin double-row female connector, serving as the core interface component for connecting the electrode array to external equipment. The exposed metal pins are the soldering terminals of the female connector intended for PCB mounting. (**D**) Photograph of the double-sided green solder mask PCB base, which contains 20 electrical connection vias and is equipped with 10 independent, electrically isolated signal interfaces on each side. (**E**) Photograph of the PCB base after soldering to the 2 × 10 pin double-row female connector. (**F**) PCB base after fixing the tungsten wire electrode tails via gold pin crimping. (**G**) Photograph of an electrode bundle being held by the electrode holder. (**H**) Schematic (**top**) and photograph (**bottom**) of the dedicated electrode holder. (**I**) Schematic (**left**) and photograph (**right**) of the gold pin.

To address the core bottlenecks of existing electrodes, and better support the acquisition of multi-region LFP signals for closed-loop neuromodulation research, we advanced prior electrode designs and developed a novel modular, silica capillary tube-based electrode. Equipped with a specialized electrode holder, this design enables efficient, precise flexible multi-region targeting, stable LFP acquisition, and simplified fabrication compatible with standard laboratory settings. The electrode can be configurable into multiple independent bundles, and each bundle accommodates multiple electrode wires, with both the number of bundles and wires per bundle customizable to experimental requirements. For the present study, we utilized a configuration of 8 bundles, 2 electrode wires per bundle, totaling 16 channels (Figure 1A), to demonstrate its performance in closed-loop neuromodulation-focused research. Among these eight bundles, the two bundles targeting the prelimbic cortex (PrL) and infralimbic cortex (IL) possess identical anteroposterior (AP) and mediolateral (ML) stereotaxic coordinates, and only differ in dorsoventral (DV) implantation depth. They are bonded side by side into a single assembly to simplify stereotaxic implantation surgery.

### 3.2. Core Components of the Multi-Region Recording Electrode System

The assembled multi-region recording electrode system is composed of three core components: a 2 × 10 pin double-row female connector (Figure 1C), eight electrode bundles encapsulated in silica capillary tube, and a printed circuit board (PCB) that acts as a relay to connect the female connector and the eight electrode bundles (Figure 1A). Each electrode bundle consisted of two tungsten wires encased in a silica capillary tube, with the wire tips extending a uniform 0.5 mm beyond the tubing (Figure 1B). This controlled tip exposure enabled stable contact with neural tissue while minimizing mechanical injury during insertion. The silica capillary tube provided both mechanical protection against wire breakage and a convenient clamping interface for precise stereotaxic implantation (Figure 1G,H). The PCB base adopted a double-sided green substrate design, with 10 output interfaces arranged on each side and 20 through-hole perforations (Figure 1D). These perforations accommodated the tail ends of tungsten wires and gold pins for signal transmission (Figure 1I). Insertion of gold pins reliably secured the tungsten wires and penetrated their insulation layer, forming a stable electrical connection without requiring pre-soldering at the wire-PCB interface (Figure 1F). The PCB output interfaces were stably connected to a commercial 2 × 10 pin double-row female connector (Figure 1E), ensuring compatibility with standard signal converters and mainstream electrophysiology recording systems.

### 3.3. Electrical Performance of the Electrodes

Impedance testing was performed on each channel of the electrodes to evaluate electrical conductivity and consistency. We tested six electrodes, and the vast majority of functional channels exhibited impedance values within the target range of 100–1000 kΩ (Table 1), with no open or short circuits detected. Longitudinal monitoring of electrode impedance at 3 months post-implantation further confirmed that the vast majority of channels maintained stable impedance values within this acceptable range throughout the chronic recording period, verifying the long-term stability of the electrode-tissue interface.

These results validated that the gold pin-assisted connection method reliably established stable, low-noise electrical contacts between tungsten wires and the PCB. The total weight of a single assembled electrode is about 0.845 ± 0.0235 g (Table 2), making it lightweight and suitable for implantation in small experimental animals, and the total cost of a single assembled electrode was only about 19.43 US dollars (Table 3), which is significantly lower than that of commercial multi-region recording electrodes.

### 3.4. Applicability Verification in Normal Rat Emotional Brain Network

**Figure 2:**
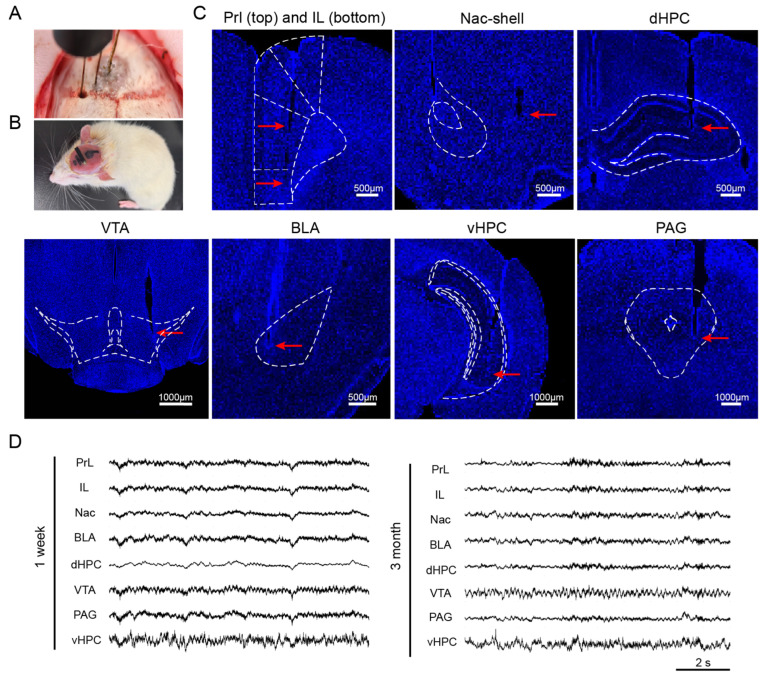
In vivo electrode implantation and electrophysiological recording in deep brain nuclei of rats. (**A**) Intraoperative scene of targeted implantation of a multi-channel electrode array into deep brain nuclei during stereotaxic surgery in a rat. (**B**) Postoperative appearance of the rat following electrode implantation surgery. (**C**) Histological verification of DAPI-stained coronal rat brain sections: red arrows mark electrode implantation trajectories within the prelimbic cortex/infralimbic cortex (PrL/IL), nucleus accumbens shell (Nac), dorsal hippocampus (dHPC), ventral tegmental area (VTA), basolateral amygdala (BLA), ventral hippocampus (vHPC), and periaqueductal gray (PAG); white dashed lines delineate the anatomical boundaries of the target nuclei. (**D**) Continuous raw electrophysiological signals recorded from each nucleus at 1 week (**left**) and 3 months (**right**) after electrode implantation.

To verify the applicability of the self-made 16-channel electrode, we synchronously recorded LFP signals from the emotional brain network in normal rats, an experimental scenario that requires flexible targeting of discrete core brain regions, which conventional fixed-layout electrodes cannot achieve. To ensure the functional relevance of recorded signals to emotional regulation, we selected brain regions that are core nodes in emotional processing and play key roles in emotional disorders such as anxiety and depression. Based on this, we chose 8 representative core brain regions, including PrL, IL, Nac, BLA, dHPC, VTA, PAG, and vHPC (Figure 2A). After 7 days of postoperative recovery, all rats exhibited natural behaviors such as grooming, exploring, and resting, with normal diet and water intake. No electrode detachment, locomotor abnormalities, or wound infection were observed, indicating good biocompatibility of the electrode (Figure 2B).

Following the completion of signal recording, rat brains were collected to verify the accuracy of the electrode implantation position. Histological examination of DAPI-stained coronal sections revealed clear implantation trajectories of each electrode bundle, which were consistent with the preset stereotaxic paths and aligned with DAPI-labeled neural nuclei boundaries (Figure 2C). For a total of 48 implantation sites (8 brain regions per rat across 6 rats), 38 sites were accurately positioned within the preset error tolerance of 50 μm, corresponding to an overall implantation accuracy of 79.2%. The remaining 10 sites showed slight deviations from the target coordinates, which may be attributed to inherent individual differences in skull and brain structure among rats, an unavoidable factor that makes complete standardization across animals challenging. During the entire experimental period, there was no signal loss or connection failure. Valid LFP signals were successfully recorded from all the target brain regions. Over the 3-month in vivo implantation and observation period, the electrode maintained stable electrical performance (Figure 2D). Collectively, these findings demonstrate that by utilizing this electrode design, we are able to achieve a relatively high implantation accuracy, stable LFP signal acquisition, and reliable long-term in vivo performance with good biocompatibility, thus supporting its suitability for preclinical multi-region LFP recording.

### 3.5. Detection of Pathophysiological Neural Activity in Acute Depression Rat Models

**Figure 3:**
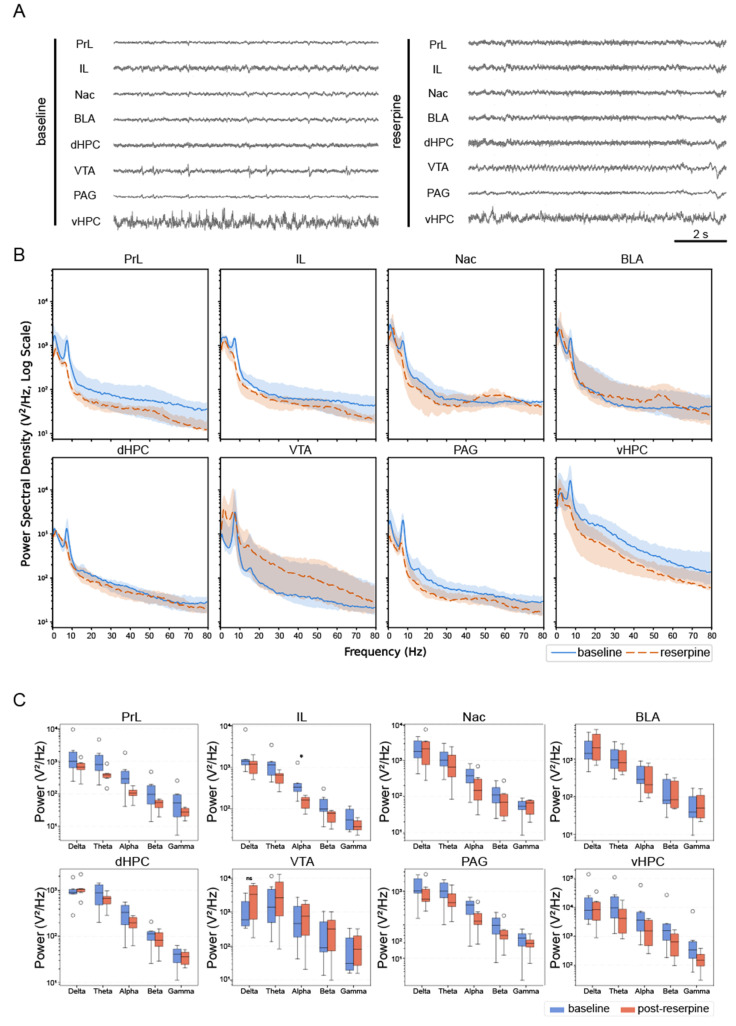
Analysis of LFP oscillatory characteristics in the emotional brain network of normal rats and reserpine-induced depression model rats. (**A**) Continuous raw LFP signals from 8 brain regions in the baseline group (**left**) and reserpine group (**right**). (**B**) Full-band (0–80 Hz) power spectral density (PSD) curves of the two groups in 8 brain regions. Solid blue: baseline group; Dashed orange: reserpine group. (**C**) Comparison of LFP power in 5 frequency bands (delta, theta, alpha, beta, gamma) across 8 brain regions (Wilcoxon signed-rank test). Blue: baseline group, red: reserpine group; left and right in each band correspond to the two groups in order. * indicates a significant difference between groups (*p* < 0.05); “ns” indicates no significant difference between groups (*p* ≥ 0.05).

To explore the electrode’s utility in detecting pathophysiological neural activity, we used reserpine to establish acute depression rat models and analyzed power spectral density (PSD) data from rats before and after administration (Figure 3A). Continuous PSD comparison revealed that both groups exhibited a typical 1/f decreasing power trend (Figure 3B), a hallmark of reliable neural electrophysiological signals, confirming the electrodes captured genuine brain activity rather than instrumental noise. PSD profile comparison revealed distinct oscillatory alterations across all 8 channels (Figure 3B). The post-reserpine group showed decreased spectral power across most channels, particularly in lower frequencies, most notably in PrL, IL and vHPC (Figure 3B). Wilcoxon signed-rank tests were performed to quantify these alterations by comparing five frequency bands (delta: 0–4 Hz, theta: 4–8 Hz, alpha: 8–13 Hz, beta: 13–30 Hz, gamma: 30–80 Hz) between baseline and post-reserpine conditions (Figure 3C). The results demonstrated that most regions showed no significant inter-group differences across bands, except for a significant reduction in alpha band power in IL of reserpine-treated rats (*, *p* < 0.05). Although these findings should be interpreted cautiously due to the small sample size (*n* ≤ 6), the results indicated that the electrode can detect subtle reserpine-associated spectral changes, while widespread non-significant differences highlight the need for larger cohorts to validate trends.

### 3.6. Machine Learning-Based Decoding of Drug-Induced Brain State Changes

**Figure 4:**
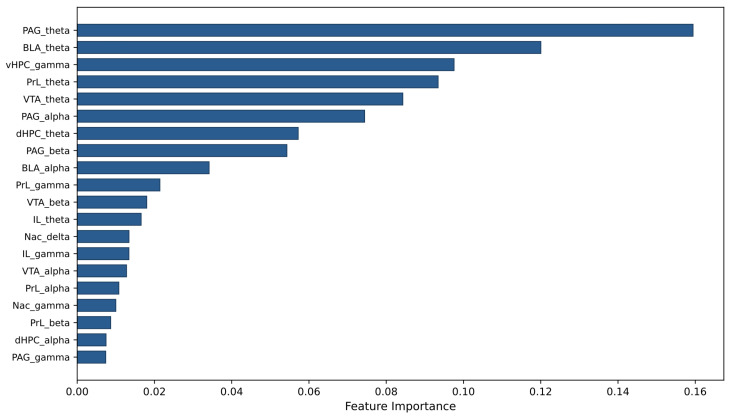
Feature importance analysis for depression state classification based on multi-region LFP oscillatory characteristics. The top 20 most important features identified by a random forest model, showing the contribution of each oscillatory feature to the classification of brain states.

Given the similarity between the multi-region recorded signals in this study and high-dimensional neural data in fields such as EEG, we adopted relevant analytical strategies from these domains and employed machine learning methods to integrate and decode this high-dimensional brain state information. A dedicated model was trained for each animal, utilizing 40 features extracted from the 8 recording channels and 5 classical frequency bands. Using a random forest classifier with five-fold cross-validation, we achieved a high average classification accuracy of 0.9637 ± 0.0145 in a representative animal (Table 4). Additionally, feature importance analysis identified and evaluated the critical neural signal features for model prediction and brain state detection. In one representative animal, the top 5 most important features were PAG_theta band, BLA_theta band, vHPC_gamma band, PrL_theta band and VTA_theta band (Figure 4). These results demonstrate a robust data processing and application pipeline for decoding drug-induced brain state changes, which lays a foundation for future closed-loop neuromodulation strategies and holds significant value for advancing translational research in neuropsychiatric disorders.

### 3.7. Multi-Brain-Region Functional Connectivity Analysis

**Figure 5:**
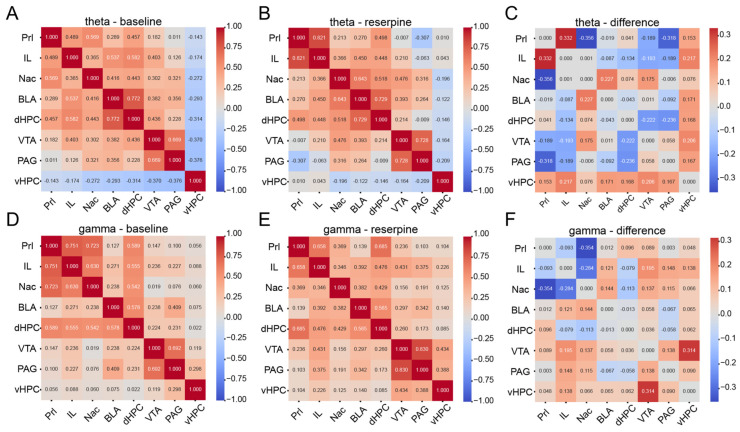
Multi-region functional connectivity analysis of local field potential signals under baseline and reserpine intervention. (**A**) Pearson correlation coefficient matrix of theta-band LFP signals in eight target brain regions (PrL, IL, Nac, BLA, dHPC, VTA, PAG, vHPC) at baseline. Color intensity represents correlation strength, and the color scale ranges from −1.0 to 1.0; (**B**) Theta-band functional connectivity matrix after reserpine treatment, using the same color scale as the baseline group for visual comparison; (**C**) Theta-band functional connectivity difference matrix (reserpine − baseline) with a color scale of −0.3 to 0.3. Red indicates enhanced connectivity and blue indicates weakened connectivity. Reserpine increases the connectivity of PrL-IL and Nac-BLA, and decreases the connectivity of PrL-Nac and dHPC-PAG; (**D**) Gamma-band functional connectivity matrix at baseline; (**E**) Gamma-band functional connectivity matrix after reserpine treatment; (**F**) Gamma-band functional connectivity difference matrix, in which the connectivity of VTA-vHPC is significantly enhanced, while the connectivity of PrL-Nac and IL-Nac is weakened.

To explore the coordinated activities of emotional brain networks under reserpine intervention, we conducted multi-region functional connectivity analysis using local field potential (LFP) signals recorded by this electrode system to further verify its brain network analysis capability. The LFP signals were filtered into five canonical frequency bands via a fourth-order zero-phase Butterworth filter, and Pearson correlation coefficients among eight target brain regions were calculated at baseline and after reserpine treatment. Averaged functional connectivity matrices and corresponding difference matrices were established to quantify drug-induced neural connection changes. Due to the limited number of animals collected, we present results from one representative rat as an illustrative example, focusing on the theta and gamma bands. The results showed that both baseline and post-reserpine functional connectivity matrices exhibited distinct, region-specific correlation profiles, with connection strengths aligning with physiologically plausible patterns (Figure 5A,B for theta; Figure 5D,E for gamma). In the theta band, reserpine induced a positive difference in PrL-IL (difference = +0.33) and Nac-BLA (difference = +0.23), but negative differences in PrL-Nac (difference = −0.36) and dHPC-PAG (difference = −0.24). In the gamma band, the most notable difference was an increase in VTA-vHPC (difference = +0.31), whereas PrL-Nac and IL-Nac showed decreased connectivity (difference = −0.35 and −0.28, respectively). These results demonstrate that the signals acquired by our electrode system can effectively support functional connectivity analysis.

## 4. Discussion

This study systematically verified the feasibility of multi-region implantation and long-term recording performance of a custom-built 16-channel modular silica capillary tube-based electrode, aiming to fill the existing gap in long-term, network-oriented multi-region brain signal recording for closed-loop research. As a representative application, we targeted the emotional brain network in rats and implanted the electrode into 8 core distributed nodes of this network. The results showed that the electrode achieved reliable implantation accuracy, maintained stable signal acquisition performance over 3-month, and could effectively capture neural activity changes and support high-precision brain state decoding. These findings confirm that the modular electrode enables robust multi-region LFP recording and represents a flexible, low-cost, and generalizable tool for investigating brain network mechanisms and facilitating preclinical closed-loop neuromodulation research.

### 4.1. Superiority of the Modular Electrode over Conventional Designs

Previous studies have established that multi-region simultaneous neural recording is essential for decoding brain network dynamics and inter-regional interactions, which cannot be fully revealed by single-region recording. However, a major bottleneck in current research lies in existing electrode strategies, which fail to reliably support such network-level signal acquisition, thereby impeding progress in closed-loop neuromodulation. Most clinically or preclinically available electrodes, including sEEG probes, silicon-based arrays, and many flexible neural electrodes, are designed for linear or fixed-layout recording, which restricts their ability to target multiple spatially discrete nodes within a distributed functional brain network [17]. In contrast, the modular silica capillary tube-based electrode in this study was specifically designed to address these pain points, while achieving an optimal balance of performance, flexibility, and cost-effectiveness. The overall implantation accuracy of 79.2% further validates the practicality of this modular design, which enables independent and flexible targeting of distributed brain regions without rigid spatial constraints.

### 4.2. Advantages of LFP Signals in Advancing Closed-Loop Neuromodulation

Unlike single-unit spikes, which often degrade over time due to gliosis, electrode micromotion, and neuronal loss during chronic implantation, local field potentials reflect population-level synaptic activity and exhibit superior long-term stability [26,27]. Such stability is critical for consistent brain state decoding and reliable closed-loop neuromodulation over extended periods. Moreover, LFP signals carry rich oscillatory information directly linked to network states and pathological dynamics, enabling more robust and generalizable brain state decoding [28]. It is for these reasons that the current study focused on stable LFP recording rather than high-resolution spike detection, as a practical and reliable strategy for brain state decoding in future closed-loop systems. More importantly, the multi-region LFP recording fills a critical technical gap left by other mainstream neuroimaging and electrophysiological techniques. Functional MRI provides high spatial resolution but suffers from poor temporal resolution, making it unsuitable for real-time feedback required by closed-loop neuromodulation [29,30]. Conversely, EEG offers high temporal resolution but is limited to superficial cortical signals with low spatial accuracy and poor access to deep brain nuclei. In contrast, the multi-region LFP approach enabled by our modular electrode achieves both high temporal resolution and precise anatomical targeting of cortical and deep brain regions, allowing faithful reconstruction of dynamic functional networks. This advantage provides a critical physiological basis for identifying network-level biomarkers and developing targeted closed-loop neuromodulation strategies.

### 4.3. Modularity, Scalability and Cost Advantages of the Proposed Electrode

A prominent advantage of the proposed electrode is its high modularity and scalability beyond the 16-channel configuration used in this study. Based on standardized commercial connectors and simple assembly procedures, the system can be readily expanded to 32 channels or higher by adjusting the number of electrode bundles and wires per bundle. The modular design allows customized configurations, including the number of electrode bundles, targeted brain regions, and channel count, to meet diverse experimental requirements. Therefore, this electrode is not restricted to the emotional network but can be widely applied to investigate various functional networks underlying neurological and psychiatric disorders, such as Parkinson’s disease, epilepsy, mood disorders, and cognitive dysfunction. Meanwhile, the use of conventional materials and simplified fabrication significantly reduces the overall cost compared with commercial silicon-based or flexible electrodes, making it affordable and accessible for routine preclinical research.

### 4.4. Integrated Pipeline for Multi-Region LFP Recording and Analysis

Beyond the electrode itself, this study established an integrated experimental and analytical pipeline covering customized electrode fabrication, multi-region stereotaxic implantation, long-term LFP recording, spectral feature extraction, functional network analysis, and machine learning-based brain state decoding. This pipeline aligns with recent research trends in neural engineering that utilize machine learning for high-dimensional neural data integration to advance closed-loop neuromodulation, BCI development, and brain mechanism research. Most existing strategies have been developed for EEG or fMRI, with limited extension to multi-region LFP signals, despite their superior spatial specificity and deep-brain accessibility. Our findings support the feasibility of transferring mature analytical methods from EEG and fMRI research (such as Granger causality analysis and microstate analysis) to multi-region LFP studies. This migration avoids the need for entirely new analytical frameworks and accelerates the interpretation of network-level neural dynamics. Future studies with larger animal cohorts can further improve cross-animal generalization and facilitate the integration of this pipeline into fully functional closed-loop neuromodulation systems.

### 4.5. Study Limitations

Several limitations of the current study should be acknowledged. First, caution should be exercised when interpreting the brain state decoding results based on machine learning. We adopted a strategy of training dedicated models for each individual animal, focusing on individual-level analysis rather than performing cross-animal generalization and validation. This is particularly notable in the feature importance analysis. Critical features were identified on an individual animal basis, and their generality across different animals was not explored. Conducting such cross-animal generalization analyses would require a larger cohort of animals, which was not the focus of the current study. Although leave-one-subject-out (LOSO) cross-validation would be ideal for assessing cross-animal generalizability, the limited sample size (*n* = 6) yields only six validation folds with highly imbalanced trial counts per fold, making LOSO estimates unstable and difficult to interpret. We therefore prioritized within-animal stratified cross-validation as the most appropriate evaluation strategy for the present dataset. Second, this study focused on validating the technical feasibility of multi-region local field potential (LFP) recording and brain state decoding, rather than developing a fully integrated closed-loop neuromodulation system. In principle, the current electrode design can be extended to support both signal recording and current pulse delivery via the same tungsten wires, analogous to previously reported approaches that enable recording and stimulation through separate channels on the same electrode [31,32]. The PCB relay design used in this study provides electrical isolation between channels, making it compatible with bipolar stimulation configurations. Future work will focus on integrating a real-time feedback controller to enable fully adaptive closed-loop neuromodulation. Although our multi-region recording approach provides a reliable neural readout, subsequent work is required to integrate recording with adaptive stimulation, optimize stimulation targets and parameters, and validate the performance of a complete closed-loop system.

## 5. Conclusions

In conclusion, this study developed and validated a low-cost (~19.43 USD/unit), modular, silica capillary tube-based 16-channel electrode enabling stable long-term multi-region LFP recording in freely moving rats. The electrode achieved 79.2% implantation accuracy across eight distributed emotional network nuclei and maintained stable signal quality over three months. Using a reserpine-induced depression model, we demonstrated that the system could detect state-specific oscillatory changes, particularly reduced alpha power in the infralimbic cortex, and that machine learning-based decoding achieved ~0.96 classification accuracy for distinguishing brain states. The modular design is highly scalable and adaptable to diverse functional networks, while the integrated analysis pipeline enables standardized spectral feature extraction and brain state decoding. Although a complete closed-loop system incorporating adaptive stimulation remains to be implemented, this work provides a practical, low-cost platform for preclinical brain network research and lays the technical groundwork for future closed-loop neuromodulation studies.

## Figures and Tables

**Table 1:** Electrode channel impedance (kOhm) across 16 channels for six electrodes.

	NO	CH1	CH2	CH3	CH4	CH5	CH6	CH7	CH8	CH9	CH10	CH11	CH12	CH13	CH14	CH15	CH16
3 day	1	264	603	497	492	364	541	415	403	318	425	717	502	464	502	601	512
	2	240	243	582	472	700	516	423	681	239	262	430	525	431	378	286	264
	3	308	373	431	352	559	464	661	612	292	342	293	332	369	383	406	450
	4	344	278	427	497	320	318	393	392	473	402	417	435	403	364	458	419
	5	120	122	119	121	167	160	223	381	161	141	141	167	106	151	238	212
	6	229	218	232	236	291	334	314	328	473	771	382	455	409	414	483	467
3 month	1	209	133	397	426	646	757	1140	511	671	622	508	505	679	171	231	97
	2	324	102	314	126	883	171	1027	193	260	131	329	220	669	132	302	119
	3	115	101	150	120	130	137	131	111	116	112	113	117	116	116	116	116
	4	246	103	114	125	389	160	210	164	256	110	141	137	293	200	229	112
	5	529	241	158	246	120	109	147	129	145	117	435	115	660	156	317	312
	6	535	250	950	269	179	299	247	119	282	116	253	115	233	242	255	107

**Table 2:** Weight of six electrodes (g).

NO	Weight (g)
1	0.88
2	0.82
3	0.84
4	0.82
5	0.85
6	0.86
Average	0.845 ± 0.0235

**Table 3:** Estimated cost breakdown for fabricating a single electrode. All costs are reported in US dollars (USD).

Name	Manufacturer	Part No.	Specification	Quantity	Total Cost (USD)
2 × 10-pin double-row female connector	Shenzhen Nanmenzi Technology Co.	1.27 mm pitch	piece	1	0.03
Custom-printed circuit board (PCB)	—	—	piece	1	0.35
Tungsten wires	California Fine Wire Co.	size 0013	mm	960	6.09
silica capillary tubes	Polymicro Technologies	TSP100170	mm	240	5.43
Silver wires	Kunshan High-Purity Electronic Materials Business Department	SsAg	mm	240	0.50
Gold pins	Creation Tech	GP-100	piece	16	7.03
Total	—		—	—	19.43

**Table 4:** Five-fold cross-validation.

Five-Fold Cross-Validation	Accuracy	Precision	Recall	F1	ROC_AUC
1	0.9883	0.9775	1	0.9886	0.9979
2	0.9532	0.9341	0.977	0.9551	0.9975
3	0.9474	0.9432	0.954	0.9486	0.9925
4	0.9591	0.9655	0.9545	0.96	0.9863
5	0.9708	1	0.9432	0.9708	0.9966
Average (mean ± SD)	0.9638 ± 0.0145	0.9641 ± 0.0237	0.9657 ± 0.0204	0.9646 ± 0.0140	0.9942 ± 0.0044

## Data Availability

The raw data supporting the conclusions of this article will be made available by the corresponding authors upon request.
